# Investigating the modulation of active preparation and passive dissipation on inhibitory control processes in the language switching paradigm

**DOI:** 10.3389/fpsyg.2023.1065268

**Published:** 2023-01-27

**Authors:** Qinfang Shen

**Affiliations:** Department of Theoretical and Applied Linguistics, University of Cambridge, Cambridge, United Kingdom

**Keywords:** language switching costs, preparation effects, bilingual language production mechanism, L2 inhibitory control, asymmetrical switching costs

## Abstract

**Introduction:**

Previous language-switching studies have received scholastic attention and the observed switching cost patterns have provided empirical evidence for bilingual language control. However, results are inconsistent as the size of and (a)symmetry in switching costs differ across studies. In addition, there are various methodological differences that go beyond stimulus differences, such as the language proficiency of the participants (the participant-level factor) and the preparation time (a task-related level factor), which might be responsible for these inconsistent results.

**Methods:**

With a focus on task-related factors, the present study was designed to examine whether and how preparation time modulates the size and (a)symmetry in switching costs by using the language-switching paradigm with cue-to-stimulus and response-to-cue intervals manipulated.

**Results:**

Replicating previous literature on language switching and task switching, a clear preparation effect was observed in all trials (stay and switch trials) for both L1 and L2. The switching costs were modulated by the cue-to-stimulus intervals, and specifically, switching costs decreased when the preparation time increased. Another intriguing finding was that even when participants were offered enough time to fully prepare for selecting the target language at the cue window, the switching costs were not completely eliminated. In terms of the passive preparation at the response-to-cue interval, switching costs could be modulated by the response-to-cue interval – the time for passive dissipation of inhibitory control applied in previous trials. The size of switching costs was clearly modulated by manipulating response-to-cue intervals and switching costs decreased as the waiting time after a naming response increased.

**Discussion:**

This study provides empirical evidence for the modulation of preparation effects on switching costs and inhibitory control mechanisms in bilingual language production.

## 1. Introduction

One of the most astonishing abilities of fluent bilinguals is that they can switch between their two languages effortlessly and seamlessly. However, it has been well documented in previous language production and comprehension studies that semantic representation activates bilinguals’ two lexicons in parallel, that is, both languages (the first language and the second language) will be activated even when the bilingual is only speaking in one of them (van Heuven et al., 1998; Costa et al., 1999; Dijkstra and van Heuven, 2002).

One important issue in this respect is why the language co-activation does not result in massive intrusions from the non-target language when speaking in the target language, for instance, previous evidence has shown that bilinguals rarely make language errors (Poulisse and Bongaerts, 1994). These findings have led Green (1998) to argue that a language control mechanism must be in place to mediate the concurrent language co-activation, which inhibits the activation of the non-target language in order to produce speech in the target language. So far, Green’s Inhibitory Control model (the IC model, hereafter) has received compelling evidence from the language switching paradigm, where participants have been asked to name objects or Arabic numbers in either their first or second language (e.g., Meuter and Allport, 1999). This naming condition leads to two types of trials: (1) the stay trial in which the naming language in the current trial is the same as the preceding trials; and (2) the switch trial in which the current response language differs from the one used in the previous trial. The typical finding is that switch trials result in slower naming latencies and more naming errors than stay trials. The naming latency difference between switch and stay trials has been referred to as so-called “language switching costs.”

However, previous language switching studies have not reached consistent conclusions regarding the size and (a) symmetry of switch costs due to various methodological differences such as different stimulus types (pictures and digits) and a variety of preparation time. Thus, the present study aimed to examine whether and how these methodological differences modulate the size of and (a) symmetry in switching costs, using a cued language switching paradigm by focusing on the preparation time of intra-and inter-trials.

## 2. Literature review

### 2.1. The general finding in the language switching paradigm: Language switching costs

In the trial-by-trial language-switching task, participants are required to name items (e.g., standardised black-and-white line drawings or Arabic digits from 1 to 9) in either their first or second language. The language in which stimuli are expected to be named depends on a colour cue (usually the colour of the background screen), varying from trial to trial. This gives rise to different types of trials. For example, in the non-switch (or stay/repeated) trial, participants name the stimulus in the same language as the one used in the preceding trial. In contrast, in the switch trial, participants name the stimulus in a different language from the one used in the preceding trial. The general finding in this context is that participants’ naming performance is impaired in the switch trial compared to the repeat trial. Specifically, switch trials result in longer naming latencies and more naming errors. The calculation of subtracting the naming latencies of switch trials from those of non-switch trials is called the “language switching cost.” These switch costs have also been found in switching paradigms that do not involve linguistic processes such as the task-switching paradigm (e.g., Rogers and Monsell, 1995; Meiran, 1996; Monsell, 2003; Kiesel et al., 2010).

The first influential study to examine the consequences of the cross-language competition and the possibility of bilingual language control was undertaken by Meuter and Allport (1999). In their study, proficient (but not balanced) bilinguals who do not have complete proficiency in two languages performed a numeral switching task, with much theoretical underpinning borrowed from task-switching theories. They were required to name Arabic numerals in either their first or second language according to colour cues. The authors hypothesised that based on the task set inertia hypothesis (Allport et al., 1994), one could predict that the dominant task that is difficulty to perform should result in larger switch costs than the non-dominant task that is easy to perform. This is because the dominant task needs to be more suppressed in order to perform the non-dominant task. As a result, when subsequently switching to the dominant task, more time and effort are needed to re-activate the dominant task. In contrast, switch costs should be smaller when switching to less dominant task, due to the less suppression exerted on the weaker task in the preceding trial. This was exactly what the authors observed.

The results showed that the naming latencies of switch trials were longer than those of stay trials and L2 switch trials, where the response language is changed from L1 to L2, resulted in shorter naming latencies than L1 switch trials, where the response language is changed from L2 to L1 did, pointing to the asymmetry in switching costs. This suggests that switching from the weaker language (e.g., L2) to the more dominant language (e.g., L1) was more costly than the other way around, resulting in an asymmetrical switching cost. The finding of an asymmetrical switching cost is interpreted as evidence of the IC model. As mentioned above, the IC model assumes that the amount of inhibitory control exerted on a language is proportional to its strength; in other words, the more dominant or stronger the language, the greater the inhibition exerted. Following this line of logic, the stronger L1 should be more suppressed when it serves as the non-target language in the L2 switch trial. As a consequence, it should take more time to overcome this inhibition when switching into the L1, that is, language re-activation becomes more difficult because of the stronger inhibition, resulting in the observed asymmetrical switch costs. Nevertheless, it should be noted that switching in such a rapid alternation between two languages required by external cue differs from natural switching in a real-life situation. This is because the former situation allows for an inhibition of the non-target language, whereas natural switching relies on a language-specific selection mechanism that does not trigger inhibitory control (Zhu et al., 2021).

Meuter and Allport (1999) also proposed that the relative proficiency levels of bilinguals’ two languages should affect the degree of switching cost asymmetry. To test this assumption, the researchers divided their participants into two groups according to their L2 proficiency levels; one group showed more L1 dominance while the other comprised relatively balanced bilinguals. It was found that the unbalanced participants exhibited asymmetrical switch costs, while the balanced group did not, which suggests that the language proficiency could modulate the asymmetry in switching costs, and further confirms the assumption that inhibition applied to an unintended language is proportional to its relative strength (Calabria et al., 2013).

Subsequent studies have replicated this result. For instance, in Costa and Santesteban (2004) study, highly proficient balanced Spanish-Catalan bilinguals were required to name pictures in a language switching paradigm in which 10 different items were presented in 950 trials (Experiment 2), with half of the responses in L1 and the other half in L2. Each picture was presented 95 times in Experiment 2. In Experiment 3, 40 new sets of pictures were employed and thus the repetition of the same picture stimuli was reduced to 23 times. The results of both Experiments 2 and 3 showed that (1) switching from L1 to L2 took the same amount of time as switching from L2 to L1, and (2) naming latencies of L1 were longer than those of L2. Thus, when participants switch between languages of a relatively similar strength, the asymmetry in switch costs should disappear due to the same amount of suppression exerted on both languages.

So far, the experimental evidence reviewed is consistent with the tenets of the IC model. However, several findings seem to be problematic. It has been shown that switching between stronger and weaker languages does not necessarily cause asymmetrical switch costs as predicted in the IC model. For instance, in Costa et al. (2006) study, when highly proficient trilinguals switched between their L1 and a much weaker L3, symmetrical but not asymmetrical switch costs were observed. These findings led the authors to argue that highly proficient bilinguals are more likely to develop a “language-specific selection” mechanism that allows them to directly select the lexical items in the intended language regardless of the competition and strength of the non-target language for speech production (Costa et al., 1999). Therefore, the inhibition of the non-target language is not necessarily required to achieve successful speech production. In contrast, for those less proficient bilinguals, the inhibitory control mechanism is still functional in the lexical production process.

Other results also seem to be more difficult to reconcile with the IC model. For instance, Philipp et al. (2007) did not find asymmetrical switch costs in participants in respect of three language pairings in a cued picture-naming task. They argued that asymmetrical switch costs were due to the persisting activation of the less dominant language instead of dominance-related inhibition. Additionally, they investigated the influence of preparation time on switching costs. Given general findings in the task-switching paradigm showing that a longer preparation time (e.g., the interval between the colour cue (used to inform participants of response language for the current trial) and stimulus onset) could reduce the switch costs because of the advanced configuration for the upcoming task (Rogers and Monsell, 1995), the same pattern of results should be replicated in the language switching paradigm. However, by manipulating the length of cue-to-stimulus intervals, the authors unexpectedly found that preparation time did not affect the asymmetry but increased the size of switching costs rather than decreasing it. As they argued, this unexpected result was due to the benefits of repetition trials, that is, participants could prepare for the non-switch trials much better than for the switch trials, thus leading to the increased switch costs.

As a modified version of the traditional switching paradigm, a cued switching paradigm has been employed in previous studies (e.g., Meiran, 1996; Costa and Santesteban, 2004; Philipp et al., 2007; Verhoef et al., 2010; Declerck et al., 2012). In this type of switching paradigm, to specify the currently required language or response, a language cue (e.g., the colour or national flag) usually precedes the stimulus, which differs from the traditional language switching paradigm in which cues and stimuli are presented simultaneously. The effect of this advanced language cue will be addressed in the following section “preparation effects in the cued switching paradigm.” Such a cued switching paradigm provides the possibility of varying the intervals between the language cue and the stimulus, which has been termed the “cue-to-stimulus” interval.

### 2.2. Preparation effects in the cued switching paradigm

In the cued language switching paradigm, the “preparation effect” refers to the finding of faster naming latencies when participants know which language needs to be named before the presence of the stimulus. In this section, I will first review compelling evidence for the preparation effect in both task-and language switching studies, and then I will give a clear distinction between the temporal preparation (generic preparation to process stimulus) and the temporal decay as an alternative interpretation for the observed preparation effect, which is an issue that has been ignored in most previous language switching studies.

Verhoef et al. (2009) assumed that the asymmetry in switch costs was not because of the inhibition of the non-response language but was instead due to the so-called “L1-repeat-benefit” effect. According to their argument, non-proficient bilinguals should suffer from strong interferences caused by the dominant L1 when naming in the L2 in both switch and stay or repeat trials. In contrast, when naming in the L1, they should suffer from the interference caused by the previous L2 naming only in the switch trial. This is because, in the L1 stay/repeat trial, L1 is always the dominant language, and thus eliminates the L2 interference effect. As such, the naming latencies in L1 stay trials should always be shorter than in any other trials, resulting in asymmetrical switch costs. Note that the stay trial and repeat trial are interchangeable in this study.

To test this assumption, unbalanced Dutch-English bilinguals were required to perform in the cued language switching paradigm in which both short and long cue-to-stimulus intervals were manipulated. Based on the previous literature, the authors predicted that the long cue-to-stimulus interval should provide the possibility for bilinguals to bias the response of the intended language due to endogenous control. This was exactly what they found: they obtained symmetrical switch costs with a long preparation time but asymmetrical switch costs with a short preparation time. Additionally, all trial conditions (e.g., the L1 and L2 switch trial conditions and the L2 stay trial condition) except the L1 repeat trial condition benefited from the long preparation time, which was consistent with the L1-repeat-benefit assumption. They attributed the benefits of preparation to the inhibition of the non-target language, thus arguing that the lack of such a benefit in L1 non-switch trials was because of a failure to activate L2. Since the non-target L2 representations were not activated in the L1 repeat trial naming, there was no need to overcome the inhibition to response, hence there was no benefit in preparation. These results also confirmed that the asymmetry in switch costs could be influenced by the preparation time, and with long cue-to-stimulus intervals, switch cost asymmetry can be fully overcome. Similarly, Costa and Santesteban (2004) tested their participants with cue-stimulus intervals manipulated between 500 ms (for short preparation) and 800 ms (for long preparation). The results showed that the size of switching costs reduced with the increase in the preparation interval, pointing to the contribution of a preparation effect.

Despite the consistent results of the preparation effect that have been reported in these studies, other studies have observed no detectable effect of preparation on switch costs (e.g., Stasenko et al., 2017), or only a modest preparation effects in some conditions (Lavric et al., 2019; Declerck et al., 2020), or even an increase in language switch costs with preparation (Philipp et al., 2007). For example, Philipp et al. (2007) observed an increase in switch costs when cue-to-stimulus intervals increased from 100 to 1,000 ms. They argued that “preparation was especially beneficial for repetition trials” compared to switch trials (p. 413), and resulted in a larger naming latency difference between stay and switch trials when a longer preparation time was given.

According to Kiesel et al. (2010), another possible interpretation for the preparation effect is the passive decay of the previous task activation or interference. In the task switching literature, increasing the between-trial interval has also been shown to reduce the switch costs independently of the active preparation (e.g., Meiran, 1996; Monsell and Mizon, 2006), possibly because a longer between-trial interval allows for the passive dissipation of the “task-set inertia” of the preceding trial. The between-trial or response-to-stimulus interval refers to the interval between the response in the preceding trial and the onset of the next cue (Kiesel et al., 2010, p. 854). Following this line of logic, in the language switching paradigm, if switching between languages is difficult because recovering from the previously inhibited language takes time, a longer decay of the previous language inhibition should reduce the switch costs. Returning to Verhoef et al. (2009) study, they had varied response-to-cue intervals due to an inter-trial latency jitter, which means that they did not rule out the effect of the passive decay of the previous inhibition on switching costs. As a consequence, the varied response-to-cue intervals may have affected the validity of Verhoef et al. (2009) experimental results.

The reduction in switch costs with advanced preparation time has been widely interpreted as “the most compelling evidence for an endogenous (“top-down”) control process of task-set reconfiguration” (Monsell, 2003, p. 1107), which comes into play before the onset of the stimulus. However, the previous literature also shows that even when sufficient advanced preparation time was given, so-called “residual” switch costs remain (e.g., Meiran, 1996, 2000; Meuter and Allport, 1999; Verbruggen et al., 2007). To interpret these findings, Rogers and Monsell (1995), Meiran (2000), and Rubinstein et al. (2001) put forward a “two-stage attentional control” model regarding the task reconfiguration processes. According to this model, task reconfiguration processes occur at two different stages. The first task-set-reconfiguration process occurs following cue onset. The general finding of the decreased switch costs with increased cue-to-stimulus intervals can be taken as evidence for this process, which has been referred to as the “endogenous component” of task-set reconfiguration (also known as “advanced reconfiguration”). “Exogenous component” of task-set reconfiguration process can only take place following stimulus onset, therefore leading to residual switch costs (Monsell, 2003).

Rogers and Monsell (1995) assumed that the preparation-benefit effect only occurred in the switch trial, and not in the stay trial. Nevertheless, there has been compelling evidence showing that language preparation is not always switch-specific. Some studies varying the preparation time also found that naming latencies reduced in non-switch trials with the increase in the preparation time. For instance, Mosca and Clahsen (2015) tested participants in a cued language paradigm in which cue-to-stimulus intervals varied from 0 to 800 ms. The results revealed that long cue-stimuli intervals reduced naming latencies in switch trials as well as in repetition trials. Furthermore, Philipp et al. (2007) study even found that the preparation effect was greater for the stay trial than for the switch trial.

Taken together, despite fruitful results in respect to the preparation effect in the task switching literature, very few language switching studies have specifically examined the effect of active preparation on language switching costs. In addition, no language switching study has examined whether and how the passive dissipation of inhibitory control modulates switching costs; that is, the effect of the response-to-cue interval on switching costs remains unclear in the current body of language switching research.

### 2.3. Global slowing of L1 in the language switching paradigm

One of the overlooked findings in the previous language switching literature is that naming latencies in L1 are always longer than in L2, even when there is no evidence for asymmetrical switching costs. That is, bilingual participants have been shown to name stimuli more slowly in their L1 than in their L2 in both repeat and switch trials (e.g., Costa et al., 2006; Christoffels et al., 2007; Gollan and Ferreira, 2009). This so-called “paradoxical language effect” has been taken as an indicator of globally inhibitory control of L1. This finding is particularly unexpected and striking, considering that in the previous literature on bilingual language production, L2 picture naming latencies have been shown to be significantly longer than those of L1 (e.g., Ivanova and Costa, 2008; Kroll et al., 2008; Hanulova et al., 2011). One of the possible accounts of the L2 naming delay is the “weaker links hypothesis” (Gollan et al., 2005, 2008). This hypothesis assumes that since the lexical items in the non-dominant language (e.g., L2) are used less often than those in the dominant language (e.g., L1), there should be weaker links between the conceptual representations and lexical forms of L2, thus leading to delayed language production in L2.

Hanulova et al. (2011) questioned whether the results of language switching studies could be extended to general bilingual contexts in which a non-mixed picture naming task is used. Non-mixed picture naming refers to pure block naming where only one type of trial is presented. This doubt is reasonable, considering that switching between two languages in a short time is certainly not a standard situation for bilinguals. However, problematic to this so-called “paradoxical naming effect” is that L1 slowing naming has not been reported in every cued language switching study (e.g., Meuter and Allport, 1999; Declerck et al., 2012). For instance, Costa and Santesteban (2004) and Costa et al. (2006) observed that proficient bilinguals only showed slower L1 naming when they also showed symmetric switch costs; that is, they did not show global slowing of L1 in the case of asymmetrical switch costs. In addition, Costa and Santesteban (2004) reported that preparation times of 0, 500 to 800 ms did not modulate this paradoxical naming effect. In addition, Declerck et al. (2012) did not observe any L1 global slowing. Taken together, previous studies on language switching reveal a complex pattern of results that are sometimes in accordance with the IC model but in other instances are not. Therefore, further studies need to be conducted to investigate how relevant experimental designs and settings modulate the pattern of language switches.

## 3. The present study

*Hypothesis 1*: Switching costs will be modulated by both active preparation of selecting the target language and passive dissipation of waiting time after the production response. In other words, the longer the preparation time, the smaller the switch costs.

*Hypothesis 2*: The switching costs will not be fully eliminated, even when longer active preparation time is given, because some components of the inhibition process will only occur after the onset of the stimuli, indicating a residual component of switch costs.

The preparation effects in the language/task switching paradigm have been interpreted as showing that a switching process takes advantage of the interval between cue and stimulus to accomplish some of its work in advance. It makes sense that when more time is given to such advanced preparation, the faster and less error-prone the naming performance becomes. Furthermore, this argument has led some of the literature on language switching and bilingual language control to argue that some component of language control processes could occur in a preparatory cue-to-stimulus interval (CSI) and the remaining components could occur following the stimulus onset (e.g., Green, 1998; Misra et al., 2012; Zhu et al., 2018). However, despite fruitful findings regarding the preparation effect in the task switching literature, very few language switching studies, except Mosca and Clahsen (2015) study, have specifically examined the effects of active preparation on switching costs. Thus, the present study was conducted in order to fill this research gap and more importantly to explore the way in which methodological differences that go beyond stimulus differences could affect the size and (a) symmetry of switching costs.

More critically, in addition to the active preparation manipulated by the cue-to-stimulus interval, there has been evidence showing that passive dissipation of the waiting time measured by the response-to-cue interval can also affect responses in task switching studies, such that longer intervals between the response and cue (of the subsequent trial) would improve performance in the next trial (e.g., Kiesel et al., 2010). This reduction in naming latencies and error rates could be due to the passive decay of the activation levels of previous trials or the dissipation of inhibitory control exerted in the last trial, which lead to smaller switching costs. However, the effect of response-to-cue intervals on switching costs has been largely under-investigated in the language switching literature. In addition, one of the methodological issues in previous language switching studies is that their response-to-cue intervals were relatively short or even left uncontrolled, for instance, varying from 1,500 to 2,300 ms in Verhoef et al. (2009) study, and from 1,000 to 1,250 ms in Fink and Goldrick (2015) study. Therefore, it remains unclear whether the passive decay of previous language interference (i.e., inhibition of the non-target language) could affect switching costs. To this end, one of the goals of this study is to investigate the effect of active preparation and passive decay of inhibition of the non-target language on language switching costs.

In the first section of this experiment, cue-to-stimuli intervals were manipulated within participants with 600 ms (shorter active preparation) and 1,400 ms (longer active preparation) cue-to-stimulus intervals, while the passive decay measured by response-to-cue intervals was kept constant at 700 ms. This research design allowed for a clear investigation of the effect of active preparation on language switching cost independent of passive interference from the previous trial. Following the same line of logic, the second section of the experiment aimed to explore the effects of passive dissipation of inhibitory control on language switching costs. The response-to-cue stimulus intervals were manipulated within participants, at 600 ms (shorter passive decay) and 1,400 ms (longer passive decay), while the cue-to-stimuli intervals were kept constant as 700 ms. This design allowed for the investigation of the effect of passive decay during waiting time on language switching costs.

For hypothesis one, faster naming responses are expected to be observed in the longer preparation condition compared to those in the shorter preparation condition. That is, the longer the cue-to-stimulus interval and response-to-cue interval, the more the switch costs decrease (i.e., through the reduction in naming latencies and error rates). A general finding in the task switching literature is that increasing the preparation time before the presentation of upcoming tasks leads to faster response latencies and to the reduction of the error rates (e.g., Meiran, 1996, 2000; Kiesel et al., 2010). With respect to the other aspect of the preparation effect – the passive decay of the inhibition of the non-relevant task – it has been found that switch costs decrease with an increasing response-to-cue interval (e.g., Koch, 2001; Koch and Allport, 2006), which is in accordance with the assumption that inhibition of the non-relevant task decays following execution of a response.

For hypothesis two, a general finding regarding the residual component of switching costs is that in spite of the fact that advanced preparation could reduce the switching cost, it never eliminates the switching costs, which suggests that endogenous control is restricted in nature and residual switch costs remain in general. Multiple interpretations have been advanced for residual switch costs in the cued task switching literature. The first account argues that these residual switch costs originate from additional stimulus-triggered processes that are insensitive to advanced preparation during the cue-to-stimulus interval (Koch and Allport, 2006). This is also put forward in the so-called two-step model of control processes in which Rogers and Monsell (1995) devised two components of control processes during task switching: (1) an endogenous reconfiguration component that occurs at the cue window and accounts for the active or advanced preparation effect (e.g., smaller switching costs with a longer cue-to-stimulus interval) and (2) an exogenous component (as triggered by the task stimulus) of the reconfiguration process. Consequently, residual switch costs can be attributed to a component of the reconfiguration process that cannot be completed until the given stimulus is presented. However, as previous scholars have argued (e.g., Allport and Wylie, 2000; Koch, 2001; Kiesel et al., 2010), the common vulnerability of this models is that it is *post hoc*, being based on the observation of the residual switch costs. Nevertheless, recent neuroimaging studies (Verhoef et al., 2010; Guo et al., 2013; Zhu et al., 2018) have provided support for the model by showing that distinct cerebral areas are involved following cue onset and stimulus onset.

The second account claims that insufficient preparation results in residual switch costs, and it should be possible to fully prepare for the upcoming task and thus eliminate residual switch costs. One of the theories in favor of this idea is the failure-to-engage (FTE) theory proposed by De Jong (2000), who holds that the residual switch cost comes from failures to engage in advanced preparation during the cue-to-stimulus interval. That is, when participants fail to engage during the preparation interval, the control system is in an “unprepared state,” and the preparation “needs to be done after the stimulus is presented” (Verbruggen et al., 2007, p. 343). One might predict that sufficient preparation should reduce residual switching costs. This idea has received support from Mayr and Kliegl (2000). Intriguingly, several papers from language switching studies have provided support for this idea by showing that with long enough cue-to-stimulus intervals, the switching costs disappeared; for example, for the 800 ms in Mosca and Clahsen (2015) study in which no switching costs were observed. However, these findings have been challenged by subsequent studies that continued to observe switching costs even when a longer preparation time was given. One possible reason for this is that those studies employed a limited number of pictures as stimuli, leading to a repetition priming effect that affected the reliability of the results. Therefore, the present study aimed to re-evaluate this issue by carefully choosing the stimuli in order to obtain a clear picture of whether the residual component of switching costs is resistant to the cue-to-stimulus interval.

## 4. Methods

### 4.1. Materials and design

In order to exclude stimulus-related effects on switching costs, a total of 164 objects were selected from the International Picture Naming Project (2005) (accessed on November 11, 2021) so that the picture stimuli were named in Chinese or English. This kind of consideration is important given the evidence that repetition priming effects could significantly reduce the size of switching costs. Nevertheless, several language switching studies have employed highly repeated Arabic number from 1 to 9 (e.g., Meuter and Allport, 1999; Philipp et al., 2007) or a limited number of pictures as stimulus (Costa and Santesteban, 2004; Mosca and Clahsen, 2015), leading to the absence of or reduction in switching costs. Furthermore, as previous language switching studies used language pairs from the same language family (i.e., Romance language and Germanic language), phonological overlap caused by cognate words can modulate language switching costs (Declerck et al., 2012). Thus, this Chinese-English language combination allows to rule out potential effect of phonological overlap on results.

Pictures with a size of 197 * 281 pixels were presented at the center of the researcher’s laptop screen (see Supplementary Appendix for the entire list of stimuli). Participants were seated approximately 60 cm from the laptop screen. Two types of coloured squares (4 cm high * 2 cm wide) served as naming cues, and the colour of the cue indicated the response language for the upcoming object: red for Chinese and blue for English. The two colours used in the present experiment are identical to those in each country’s flag, making it easy for participants to retrieve the corresponding language through the colour cues. This consideration enables reduction of the effect of the cue mapping process on switching costs. Moreover, this design leads to two types of trial: (1) non-switch trial, in which the response language of the current trial is the same as that in the previous trial, and (2) switch trials, in which the response language of the current trial is different from that named in the preceding trial. There were 164 trials in total, which were divided into four conditional blocks with balanced number of stay trials and switch trails in each condition. There were an equal number of language switches and repetitions in each condition. The first trial was a null switch trial, and therefore there were 20 stay trials and 20 switch trials in each condition. Trial sequence can be found in Supplementary Appendix.

In the active preparation condition, the pictural stimulus was presented in the blocked condition in which the cue-to-stimulus intervals were manipulated within-subjects, varying from 600 to 1,400 ms. Specifically, in the short-preparation block, after the presentation of a fixation cross for 400 ms and the blank screen for 300 ms, the colour cue was presented for 600 ms, and an object was then presented and remained visible for 1,300 ms during which participants were required to provide a naming response. In the long-preparation block, after the presentation of the colour cue for 600 ms, a blank screen appeared for 800 ms, followed by the naming object. Critically, the response-to-cue interval was kept constant at 700 ms. Following the same line of logic, in the passive preparation condition, the cue-to-stimulus interval was kept constant at 700 ms, while the response-to-cue interval varied from 600 to 1,400 ms in the blocked condition; these were the short-decay and long-decay blocks, respectively. In summary, there were four types of blocks: (1) short-preparation block, (2) long-preparation block, (3) short-decay block, and (4) long-decay block, and each block comprised 40 trials (with 20 switch trials and 20 stay trials). The block order was counter-balanced across participants, but the trial sequence in each block was kept fixed across participants. The pictorial stimuli were not always presented in a particular condition, and a quarter of pictures were presented in the short-preparation block, another quarter in the long-preparation block, a quarter in the short-decay block, and the last quarter in the long-decay preparation block. Experimental paradigm can be found in Supplementary Appendix.

In addition, another non-preparation block served as the practice block in which Arabic digits from 1 to 9 were employed as stimuli. There were 40 trials in the practice block, and the number of switch and stay trials was balanced. In the no-preparation block, a fixation cross “+” appeared for 400 ms, followed by a blank screen for 300 ms, and then the object to be named and colour cue were presented simultaneously and remained visible on the screen for 1,300 ms, during which participants were required to provide a response. Then the fixation across “+” appeared again, indicating the start of the next trial. The aim of this practice block was to familiarize the participants with the experiment and the voice-key.

### 4.2. Participants

A total of 20 participants who were postgraduate students at British universities were recruited to participate (13 males and 7 females, mean age = 25.3). Participants were all right-handed and had normal or corrected to normal vision. Before the experiment, one-to-one interviews were conducted with participants to ask them their English proficiencies *via* self-reporting and their language dominance. They reported Chinese as their stronger first language (L1) and English as their weaker second language (L2). All participants believed that they were proficient Chinese-English bilinguals, which can also be shown by their C2 level in Common European Framework Reference as their IELTs scores were higher than 7.5. Despite the fact that they started learning English at different ages (ranging from 3 to 12 years old; mean age = 8.1), all 20 participants received formal English training from their junior high school. Finally, participants were paid (4 GBP) as compensation.

### 4.3. Procedure

Participants were tested individually in a quiet room, and they were seated approximately 40 cm from the laptop screen. Before the experiment, participants were required to sign a Participant Consent Form. Verbal instructions were then given to them, explaining that they had to name the picture stimulus on the screen as quickly and accurately as possible in either their L1 or L2 according to the colour cue.

Following the procedure of previous language switching experiment (e.g., Meuter and Allport, 1999; Costa and Santesteban, 2004; Costa et al., 2006; Philipp et al., 2007), before the formal experiment, participants were also required to name each picture stimulus both in Chinese and English without time pressure and were given the correct name of each object in the case of an error. In addition, to familiarize the participants with the experiment and the voice key, they proceeded with a practice block in which no preparation time was offered. However, it should be noted extensive and long-time language switching training before the experiment would facilitate participants’ inhibitory control and their performance in the language switching task (Liu et al., 2019).

During the experiment, written instructions were presented on the screen in Chinese. Next, each trial started with a fixation cross (“+”), followed by a blank screen. Then a red or blue square appeared on the screen for 600 ms as the language cue, immediately after which picture stimuli were presented. The stimuli remained on the screen until the voice key was activated, during which participants’ naming latencies were recorded using a Microsoft Sound Mapper connected to the laptop. Then, the next fixation cross appeared, indicating the start of the next trial. Participants were given four-minute breaks between blocks, although they were allowed to skip these if they wished. The whole experiment took approximately 30 min to complete.

### 4.4. Apparatus

The experiment was conducted using a laptop running a Microsoft Windows 10 operating system. Stimulus presentation and data collection were set out using SuperLab 6.0 software (Cedrus Corp). Naming responses were collected and recorded using an Input Microsoft Sound Mapper that measured from the display of the target stimulus to the speech onset of the vocal responses. The author sat next to participants to observe naming errors such as false triggering and incorrect naming responses.

### 4.5. Data coding

The first trial in each condition was coded as a null switch trial and thus excluded from subsequent analyses. In addition, naming responses beyond the response interval (1,300 ms) or less than 600 ms, during which the microphone was mis-triggered (e.g., by stuttering or coughing) were excluded from the data analysis (4.1% of the data). Naming errors here refer to incorrect naming responses and the inappropriate response language.

The dependent variables were participants’ naming latencies (RTs in ms) and accuracy rates (in percentage). The within-subject independent variables were the preparation type (short preparation, long preparation, short decay, and long decay), the response language (Chinese vs. English), and the language transition type (switch vs. repetition trials). The mean correct response latencies (RT) and accuracy rate data were analysed separately using analysis of variance (ANOVA) run in IBM SPSS Statistics (SPSS Inc. Released 2007. SPSS for Windows, Version 16.0. Chicago, SPSS Inc).

## 5. Results

### 5.1. Active preparation: Short vs. long cue-to-stimulus interval (600 vs. 1,400 ms)

The purpose of this comparison is to investigate the way in which preparation time modulates bilingual inhibitory control *via* the manipulation of cue-to-stimulus intervals in the cued language switching paradigm. A 2 (response language: Chinese vs. English) * 2 (transition type: switch and repeat trials) * 2 (preparation type: short vs. long cue-to-stimulus intervals) repeated measures analysis of variance by participants was performed on the naming latencies and accuracy rates.

**Figure 1:**
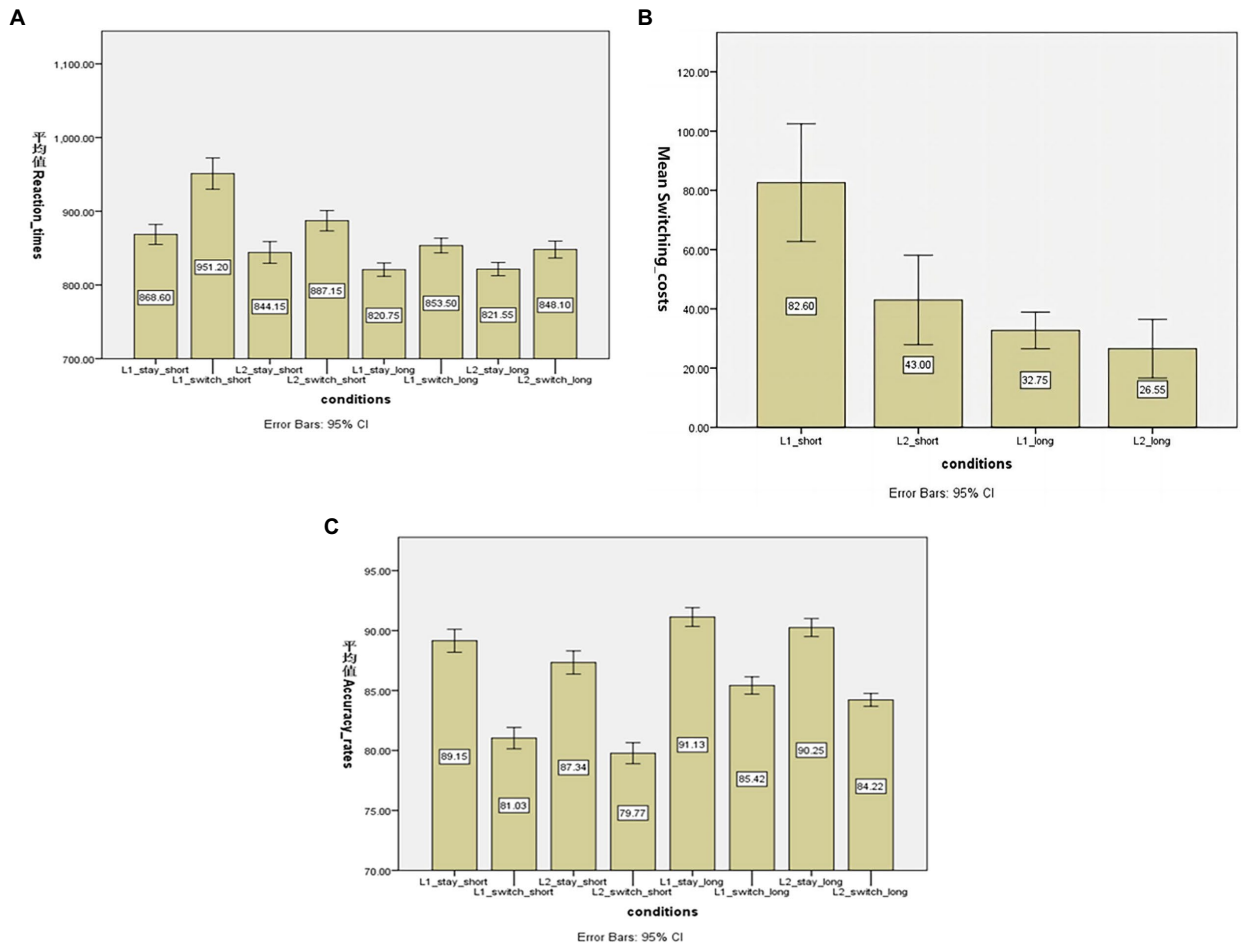
**(A)** Mean reaction times (in ms) of stay and switch trials across two preparation conditions (short vs. long) in the experiment. **(B)** Switching costs (in ms) as a function of response languages (Chinese vs. English) and preparation type (short vs. long preparation). **(C)** Mean accuracy rates (in percentage) of stay and switch trials across two preparation conditions (short vs. long) in the experiment. Error bars show 95% confidence intervals. L1, first language; L2, second language; Short, short preparation block; long, long preparation block. 平均值 (ping jun zhi): mean.

Table 1 presents mean naming latencies and accuracy rates in the different experimental conditions. First, as shown in Figure 1A that presents naming latencies in each experiment condition, the RT analysis revealed a statistically significant effect of the cue-to-stimulus interval (893 vs. 836 ms in short and long preparation conditions, respectively), *F*(1,19) = 126.732, *p* < 0.01, MSE = 1,034. 487, ηp^2^ = 0.870, reflecting overall shorter naming latencies when the length of the cue-to-stimulus increased. Second, there was also a significant main effect of transition type (841 vs. 889 ms in non-switch and switch trials, respectively), *F*(1,19) = 167. 554, *p* < 0.01, MSE = 546.028, ηp^2^ = 0.898, indicating the overall impaired performance on switch trials as compared to repeat trials, and pointing to the switching costs. In addition, it is critical that there is a clear interaction effect between the transition type and preparation type (switching costs: 63 vs. 30 ms in short and long preparation conditions, respectively, as shown in Figure 1B), *F*(1,19) = 18.912, *p* < 0.01, MSE = 567.138, ηp^2^ = 0.499, suggesting that switch trials benefited more when the active preparation time increased. This further points to the active preparation effect on the size of switch costs and reveals that if the cue-to-stimulus interval lengthens, the size of switching costs will become smaller. Moreover, it is interesting to see that even though the participants were given a long enough time to prepare, the switching costs (30 ms in the long preparation condition) were not eliminated, which is at odds with a previous study in which Mosca and Clahsen (2015) did not observe any switch costs when the cue-to-stimulus interval was 800 ms which is much smaller than that in the long preparation condition in this experiment. The current result also provides compelling evidence for residual switch costs and the argument that the exogenous component of the inhibitory control process can only take place after the presentation of the stimulus.

**Table 1:** RTs in ms and accuracy rates in percentage (standard deviations in brackets) in short and long preparation conditions.

	L1 (Chinese)		L2 (English)	
	Stay trial	Switch trial	Stay trial	Switch trial
Short preparation	868 ms (29)	951 ms (45)	844 ms (31)	887 ms (29)
	89.14% (2.0)	81.03% (1.9)	87.34% (2.1)	79.77% (1.9)
Long preparation	820 ms (19)	853 ms (21)	821 ms (19)	848 ms (24)
	91.13% (1.7)	85.41% (1.5)	90.25% (1.6)	84.22% (1.1)

In terms of the (a) symmetry of the switch costs, there was a two-way interaction effect between the response language and transition type, *F*(1,19) = 30. 993, *p* < 0.01, MSE = 169.205, ηp^2^ = 0.620, suggesting that switching costs were asymmetrical between L1 and L2, and specifically, the switching costs were larger in L1 than in L2 (see Figure 1B for the switching costs across two preparation conditions). Furthermore, there was a three-way interaction effect among the response language, transition type and preparation type, *F*(1,19) = 22.142, *p* < 0.01, MSE = 125.953, ηp^2^ = 0.58, which suggests that the asymmetry in switching costs varies across different preparation types. In the short-preparation condition, paired sample t-test analysis showed that the switch costs of L1 were significantly larger than those of L2 (82.6 vs. 43 ms), *t*(19) = 6.187, *p* < 0.01, showing the asymmetry of switch costs. By contrast, in the long-preparation condition, the switch costs of L1 were not significantly larger than those of L2 (32.7 vs. 26.5 ms, *p* > 0.05). Thus, it can be argued that with a long enough preparation time, the switching costs will become symmetrical between L1 and L2.

In the analysis of accuracy rates (in percentage, see Figure 1C), the switch cost effect was reliably observed here: switch trials resulted in more errors than non-switch trials, with 82.6% accuracy rates of switch trials and 89.46% accuracy of non-switch trials, *F*(1,19) = 571. 607, *p* < 0.01, MSE = 1880.607, ηp^2^ = 0.968. In addition, there was a preparation effect in the accuracy rates analysis, *F*(1,19) = 111.543, *p* < 0.01, MSE = 470.870, ηp^2^ = 0.854, which means that the accuracy rates increased with an increase in the cue-to-stimulus interval (87.7% for the long preparation block and 84.3% for the short one). Moreover, a significant effect was also observed in the language variables: L2 led to slightly more errors than L1 with 86.70% for L1 and 85.4% for L2 *F*(1,19) = 35.138, *p* < 0.01, MSE = 66.255, ηp^2^ = 0.649. However, two-way interaction effects such as response language * transition type, p > 0.05, *F*(1,19) =0.045, response language * preparation type, *p* > 0.05, *F*(1,19) = 1.02, and three-way interaction effects, *p* > 0.05, *F*(1,19) = 0.417, did not reach a significant level. However, there was a two-way interaction effect for transition type and preparation type, *F*(1,19) = 13.485, *p* < 0.05, MSE = 36.868, ηp^2^ = 0.415, which is in line with the observation of the RT analysis that switching costs became smaller when the active preparation time increased.

### 5.2. Analysis

First, consistent with previous studies on language switching, a “global slow L1 naming” phenomenon was observed in the RT data (but not in the accuracy rates); namely, naming latencies were reliably longer for L1 than for L2, which has been referred to as a “paradoxical language effect” (e.g., Christoffels et al., 2007; Verhoef et al., 2009). At first glance, one could argue that this result seems to contradict a typical observation in the bilingual picture naming literature in which naming latencies of L2 are slower than those for L1. However, as Kroll et al. (2008) argued, this paradoxical language effect has uniquely been found in a mixed-language context in which the participants are required to switch from one language to the other quickly, and is believed to be the result of bias in respect of L2. That is, this effect could be attributed to the additional costs exerted to globally inhibit the dominant L1 to facilitate naming in the weaker L2 (see also Costa and Santesteban, 2004 for a similar conclusion). One of the controversial findings is the presence of a preparation benefit in the L1 non-switch trials, and this was indeed observed in the current research, from 869 ms in the short preparation block to 821 ms in the longer one. This result is, however, at odds with Verhoef et al. (2009) who found that L1 non-switch trials do not benefit from a longer preparation time.

In line with previous studies on language switching with unbalanced bilinguals (e.g., Meuter and Allport, 1999; Costa and Santesteban, 2004; Philipp et al., 2007), the results of this study clearly show asymmetrical switching costs. More specifically, significant switching costs were observed in both L1 and L2, and the switching costs in L1 were significantly larger than those in L2. This pattern of results is consistent with the IC model (Green, 1998), demonstrating that the presence of switching costs is due to the effort to overcome the inhibition of the previous non-target language. In addition, the observation of asymmetric switching costs in the present experiment provides compelling evidence for the other assumption of the IC model that the magnitude of inhibition exerted on the non-target language is dependent on the proficiency level (or dominance) of that language and that is why the dominant language such as L1 is inhibited to a larger extent and overcoming such a suppression takes longer as compared to recovering from the suppression of L2.

More critically, it was observed that in the analysis of the RT data, the magnitude of the switching costs was clearly modulated by the cue-to-stimulus interval; that is, the switching costs reduced when the preparation time increased, i.e., from 63 ms in the short preparation block to 30 ms in the longer block. This is consistent with the observations of Costa and Santesteban (2004) and Verhoef et al. (2009) studies (see Mosca and Clahsen, 2015 for similar findings). Coupled with those previous findings, the results of the current study suggest that longer cue-to-stimulus intervals allow bilinguals to overcome at least some part (but not all) of the inhibition of the non-target language. Another important finding is that despite longer cue-to-stimulus intervals being offered to participants to prepare (i.e., 1,400 ms in the longer preparation block), the switching costs did not completely disappear (i.e., 24 ms for the long preparation condition). This is at odds with Mosca and Clahsen (2015) study, in which no switching costs were observed when the participants were given 800 ms to prepare. Given the findings that repeated presentation of a stimulus helps to reduce the switch costs in the language switching task, it can be argued that the repetition priming effect caused by the methodological differences between the current study and Mosca and Clahsen’s study, in which a limited number of (eight) pictures were repeatedly used as stimuli, could explain why there were no switching costs in their study. Furthermore, the smaller switch costs observed in the long preparation block might refer to what previous task switching researchers have referred to as “residual switch costs,” which are immune to elimination by the further lengthening of the preparation interval (e.g., De Jong, 2000; Schneider, 2016).

As argued earlier, these residual switching costs can be interpreted by Rogers and Monsell (1995) account of endogenous and exogenous control processes. According to the authors, the endogenous control process is defined as adopting “task-sets in advance of the stimulus, and without foreknowledge of the stimulus identity” (p. 208), and it is considered as a top-down and intentional process driven by internal goals, wills and intentions. Following this logic, it can be argued that the cue-to-stimulus interval in the language switching paradigm allows only the endogenous component of preparation to take place before the presentation of the stimulus. However, the other part of attentional control – the exogenous control process – is more like a bottom-up and non-intentional process that is activated by an external stimulus (Verhoef et al., 2009), and thus this component of inhibitory control will not benefit from the advanced preparation effect achieved by the manipulation of the cue-to-stimulus interval. Consequently, it can be argued that no matter how long it takes to prepare before the stimulus, the switching costs can never be eliminated as the exogenous control process can only be overcome after the stimulus onset.

In addition to the naming latencies, the analysis of accuracy rates also reveals the same trend of switching cost, i.e., that switch trials resulted in more errors than non-switch trials in both L1 and L2, pointing to the inhibition of the non-target language as suggested by the IC model. A significant reduction in switch costs was found when a longer preparation time was given because participants made fewer errors as the preparation time increased. It can therefore be argued that a longer preparation time is helpful in reducing the error rates in the language switching process. In addition, unlike in the RT data, the switch costs observed from accuracy rates seem to be symmetric in the long preparation condition. Nevertheless, given that most previous studies only focus on the analysis of RT data (e.g., Meuter and Allport, 1999; Verhoef et al., 2009; Ma et al., 2015) and do not recognize the value of accuracy (or error) rates, the current study thus follows this trend and puts the main emphasis on interpretation of the RT data.

It is interesting to find that the asymmetry in switching costs can be modulated by the cue-to-stimulus interval as it was found that switching costs were comparable for L1 and L2 in the long preparation block, which is consistent with Verhoef et al. (2009) study in which the authors reported symmetrical switching costs when the longer cue-to-stimulus intervals were 1,500 ms. By contrast, this seems to be at odds with Fink and Goldrick (2015) study, in which they found asymmetrical switching costs when the cue-to-stimulus intervals were spread across 0, 750, and 1,500 ms. Considering the same cue-to-stimulus intervals among these two studies, it is thus impossible for the manipulations of preparation time to make a difference to the asymmetry in switching costs. However, notably, participants in Verhoef et al. (2009) study were highly proficient Dutch-English bilinguals who started learning English at a very young age, which is sharply different from those in Fink and Goldrick (2015) study who had a very low proficiency level. As aforementioned, the participants in the current study have been studying in English-speaking environment for years, which might have provided them with more opportunities to practice their L2 to reach a high proficiency level.

It was also found that both switch and repeat trials benefited from the preparation time, which challenges the idea of a “switch-specific preparation process” proposed by Rogers and Monsell (1995). As they suggested, switching costs can be attributed to the task-set-reconfiguration processes that occur in switch trials but are not specifically required in stay trials. Consequently, prolonging cue-to-stimulus intervals would facilitate the endogenous component of task-set reconfiguration, leading to a reduction in switching costs. Apparently, what was observed in the current study does not support this assumption since the preparation benefit effect was not specific to switch trials. Monsell et al., accounts of switching costs have also been questioned by other studies on task switching that varied preparation time. These studies found that RTs decreased in repetition trials with an increase in preparation time (e.g., De Jong, 2000; Nieuwenhuis and Monsell, 2002; Koch and Allport, 2006; Kiesel et al., 2010), which provides compelling evidence for preparation in non-switch trials. Furthermore, in spite of the “general preparation process” for both types of trials, switch trials benefited more from the longer preparation time than the repetition trials did, leading to the reduction in switching costs. As argued in the IC model (Green, 1998), switching costs are attributed to the extra time needed to recover from the previously inhibited non-target language in the switch trials. This interpretation aligns more with Allport et al. (1994) argument that switching costs stem from an inertial interference from the previous task set. The purpose of this experiment, however, was not to try to explore the cause of the switching costs and the correlation between task switching and language switching. Instead, it aimed to provide a more comprehensive picture of the preparation effect.

### 5.3. Passive preparation: Short vs. long response-to-cue intervals (600 vs. 1,400 ms)

The purpose of this comparison is to investigate the influence of passive decay of the inhibition of the non-target language on switching costs through the manipulation of the response-to-cue interval in the cued language switching paradigm. A 2 (response language: Chinese vs. English) * 2 (transition type: stay and switch trials) * 2 (preparation type: short vs. long response-to-cue intervals) repeated measures analysis of variance was performed on the naming latencies and accuracy rates. The ANOVA analyses were by-participants.

In the cued language switching paradigm, participants are required to wait after a naming response until the next language cue shows. The interval between the naming response and the next language cue has been referred to as the response-to-cue interval. Previous studies using a task switching paradigm have found that the switch costs reduce as the response-to-cue intervals increase when the cue is either presented prior to or simultaneously with a stimulus. This reveals that increasing the waiting time after a response could allow participants to overcome the exogenous (or residual) component of inhibition between trials. Following the same line of logic, it can be hypothesised that response-to-cue intervals could affect inhibitory control in bilingual language production. It should be noted that this effect has not been investigated in previous language switching studies.

**Figure 2:**
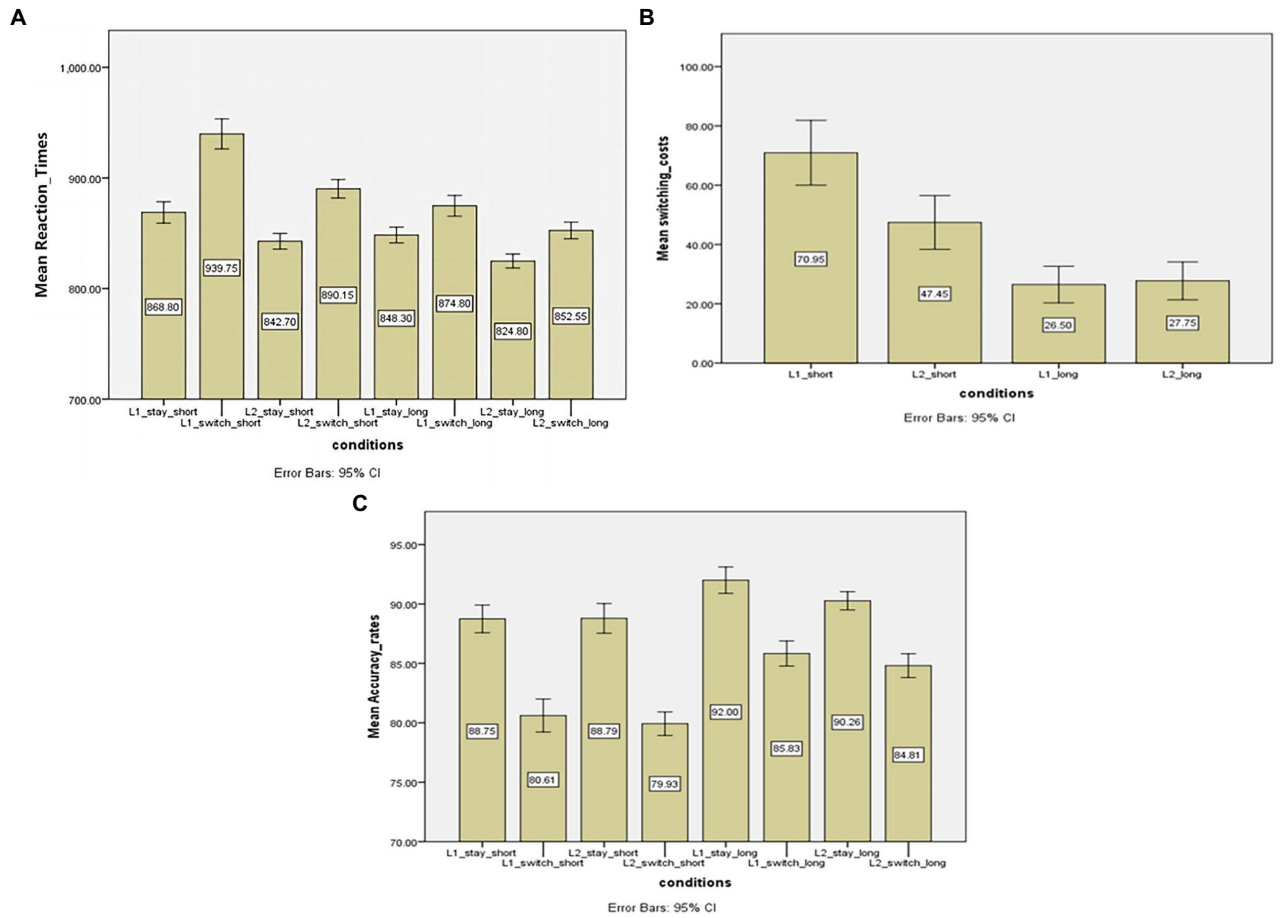
**(A)** Mean reaction times (ms) of stay and switch trials across two dissipation time conditions (short vs. long). **(B)** Switching costs (ms) across two dissipation types (short vs. long) in the experiment. Switching costs (in ms) as a function of response language (Chinese vs. English) and preparation type (short vs. long decay). **(C)** Mean accuracy rates of stay and switch trials across two dissipation time conditions (short vs. long). Error bars show 95% confidence intervals. L1, first language; L2, second language; Short, short decay condition; Long, long decay condition. 平均值 (ping jun zhi) = mean.

Table 2 presents mean naming latencies and accuracy rates across different passive preparation conditions. First, as shown in Figure 2A that presents naming latencies in each experiment condition, the RT analysis revealed a significant passive preparation effect reflected by response-to-cue intervals [*F*(1,19) = 105. 812, *p* < 0.001, MSE = 541.214, ηp^2^ = 0.848] with 885 ms and 847 ms in short and long response-to-cue conditions, respectively. This result shows the overall shorter naming latencies when the response-to-cue intervals increased. Secondly, there was also a significant effect of transition type [*F*(1,19) = 581. 606, *p* < 0.001, MSE = 117.380, ηp^2^ = 0.968] with repeat trials of 845 ms and switch trials of 887 ms, respectively. This shows an overall impaired performance in switch trials, pointing to the observation of switching costs. In addition, response language also reveals a significant effect, *F*(1,19) = 60.050, *p* < 0.001, MSE = 614.072, ηp^2^ = 0.760, suggesting that naming times were longer in L1 than in L2. So far, these results are congruent with those reported in the first comparison of active preparation effects.

**Table 2:** RTs in ms and accuracy rates in percentage (standard deviations in brackets) in short and long decay conditions.

	L1 (Chinese)		L2 (English)	
	Stay trial	Switch trial	Stay trial	Switch trial
Short RCIs	868 ms (20.7)	939 ms (29.1)	842 ms (15.2)	890 ms (17.9)
	88.75% (2.5)	80.61% (3.0)	88.79% (2.7)	79.92% (2.1)
Long RCIs	848 ms (15.1)	874 ms (20.0)	824 ms (13.5)	852 ms (16.1)
	92.00% (2.4)	85.83% (2.3)	90.26% (1.6)	84.80% (2.1)

It is important to highlight that the two-way interaction effect between transition type and preparation type was significant, *F*(1,19) = 65.270, *p* < 0.001, MSE = 196.085, ηp^2^ = 0.775, suggesting that naming latencies of switch trials decreased more than those of repeat trials as response-to-cue intervals increased, which thus points to a reduction in switching costs with lengthening the passive decay interval (see Figure 2B for switching costs in different trials). Together, this pattern of data reveals that switch costs can be modulated by the dissipation time and decreased reliably as the response-to-cue intervals increased, with switch costs of 59 ms in the short preparation block and switch costs of 24 ms in the longer one. Another important finding is that the switch costs could not be eliminated by the passive preparation effect as suggested by those in the longer dissipation time block. Additionally, other two-way interactions of language * transition type, *F*(1,19) = 8.148, *p* = 0.01, MSE = 151.893, ηp^2^ = 0.300, and language * preparation type, *F*(1,19) = 6.144, *p* = 0.023, MSE = 365.006, ηp^2^ = 0.244, were also significant, suggesting that the switching costs were larger in L1 than in L2. In a similar vein, the three-way interaction between language, transition type and preparation type was also significant, *F*(1,19) = 9.984, *p* < 0.01, MSE = 153.380, ηp^2^ = 0.344.

In order to further investigate the effect of the dissipation time on the (a) symmetry of switching costs, separate t-tests were performed. Figure 2B demonstrates the switching costs in L1 and L2 across two passive dissipation time conditions. The results show that the switching costs were asymmetrical in the short preparation block, with 70 ms for the L1 and 47 ms for the L2, *t*(19) = 3.350, *p* < 0.01. By contrast, in the long preparation condition, even though the switch costs of L2 were relatively larger than those for L1, i.e., 28 vs. 26 ms, there was no statistical difference between them; t (19) = 0.363, *p* > 0.05. Thus, it can be safely argued that the switch costs will become symmetrical with long enough response-to-cue intervals.

In the analysis of accuracy rates (see Figure 2C for the results of the accuracy rates in each condition), the main effect of the variable “Transition type” was significant [*F*(1,19) = 344.908, *p* < 0.01, MSE = 2048.190, ηp^2^ = 0.948], which indicates that switch trials resulted in more errors than non-switch trials (89.95% for non-switch trials and 82.80% for switch trials). A reliable preparation effect was also observed here: *F*(1,19) = 109.439, *p* < 0.01, MSE = 550.045, ηp^2^ = 0.852, revealing that participants made more errors (84.51%) when they were given a shorter time to prepare compared to a longer preparation time (88. 22%). There was also a main effect of the variable “response language,” *F*(1,19) = 5.065, *p* < 0.05, MSE = 28.968, ηp^2^ = 0.210, which shows that participants made fewer errors when they performed in L1 with an 86.80% of accuracy rate than in L2 (85.94%). More importantly, there was a two-way interaction effect of “Transition type” and “Preparation type,” *F*(1,19) = 16.625, *p* < 0.01, MSE = 72.227, ηp^2^ = 0.467, demonstrating that the magnitudes of the difference in accuracy rates for the switch and non-switch trials were different for two preparation conditions, and specifically, the accuracy rate difference was smaller in the long preparation condition than that in the short one. This is consistent with the naming latency analysis in which the switch costs become smaller as the preparation time increased.

### 5.4. Analysis

By manipulating the response-to-cue intervals, in this comparison, the influence of passive decay of previous inhibitory control on the magnitude and (a) symmetry of switching costs was investigated. In accordance with previous studies on language switching, the results are discussed mainly based on the RT data.

The analysis of the RT data revealed that switching costs were present in both L1 and L2, that is, switch trials led to longer naming latencies than repeat trials did in both languages. Further, the magnitude of switching costs was clearly modulated by manipulating response-to-cue intervals, and specifically, switching costs decreased as the waiting time after a naming response increased. This result is congruent with the task switching literature that demonstrates that inhibitory control declines over time after a task response has been made (e.g., Rogers and Monsell, 1995; Nieuwenhuis and Monsell, 2002). Therefore, in the language switching domain, it can be argued that inhibitory control of the non-target language dissipates over time after the target language is named. However, a reversed preparation effect was observed in the stay trials as the reaction times became longer with longer response-to-cue intervals allowing for more inhibition to dissipate, which deserves further investigation in future work.

Following this logic, this argument presents the possibility that with ample waiting time after a naming response, this kind of inhibitory control of the non-target language – the exogenous control process – could dissipate entirely. This is certainly possible, given that the cue-to-stimulus intervals could help to fully overcome the endogenous control as suggested by the previous task-switching literature. Consequently, one could argue that the longer dissipation time in the present experiment (i.e., 1,400 ms) was sufficient to eliminate the exogenous control exerted on the non-target language, and thus switching costs should disappear in the longer dissipation block. However, it needs to be noted that the cue-to-stimulus interval was kept constant at 700 ms in the passive decay condition, which was found to be insufficient to overcome the endogenous component of control since the switching costs decreased as the active preparation time (as indexed by cue-to-stimulus intervals) increased to 1,400 ms. Therefore, this leads to one possibility that those “unfinished” or “un-overcome” parts of endogenous control at the cue window of 700 ms might continue to operate after the stimulus onset, and thus this compound effect makes the switching cost more resistant to dissipation time. Based on these hypotheses, future research on preparation effects in the language switching domain needs to determine whether lengthening the active preparation time and passive dissipation time could allow recovery from the inhibitory control processes completely, thus leading to no switching costs.

In addition, a reliable asymmetry in switching costs was observed in the short dissipation time condition. Specifically, the switching costs for the L1 were larger than those for L2, which is similar to the findings for the active preparation effect. This illustrates that more inhibition of the dominant L1 was exerted than that of L2 during bilingual language production (e.g., Meuter and Allport, 1999; Costa and Santesteban, 2004; Philipp et al., 2007), thus requiring a longer time to dissipate this suppression after a naming response in L2. Similar to the observation of the active preparation, the asymmetry in the switching costs was affected by the response-to-cue interval. That is, the switching costs became symmetrical when the response-to-cue intervals were lengthened from 700 to 1,400 ms. Thus, it can be argued that a longer waiting time for passive decay of the previous inhibition of L1 enables reduction of the need for the larger extent of inhibition of L1. However, the mechanism of how the longer dissipation time enables reduction of the larger amount of inhibition on L1 remains unclear.

Finally, the fact that switching costs can be modulated by both cue-to-stimulus intervals and response-to-cue intervals provides support for Green (1998) hypothesis that inhibitory control might occur at two stages: (1) the language task schema stage following the cue onset, and (2) the lemma selection stage following the stimulus onset.

## 6. Discussion and conclusion

The present study investigated the influence of preparation effects on bilingual control in the language switching process. The logic behind this that previous literature on language switching has constantly observed different patterns of switching costs, either absent or present, asymmetrical or symmetrical, which has led the author to argue that methodological differences - preparation differences, might be responsible for such an inconsistent pattern of findings. To this end, varied active preparation times and passive decay times were manipulated to investigate how switching costs could be modulated by two types of preparation effects: (1) active preparation for selecting the target language following the cue onset, and (2) passive preparation for the passive dissipation of inhibition of the non-target language following the response. Specifically, the manipulation of cue-to-stimulus intervals could show how active preparation to select the target language at the cue window affects the inhibitory control process. In a similar vein, the manipulation of response-to-cue intervals could allow for explaining how inhibitory control dissipates over time after a naming response at the stimulus window.

Replicating previous literature on language switching (e.g., Costa and Santesteban, 2004; Costa et al., 2006; Philipp et al., 2007; Verhoef et al., 2009, 2010; Mosca and Clahsen, 2015) and task switching (e.g., Kiesel et al., 2010), a clear preparation effect was observed in all trials (stay and switch trials) for both L1 and L2. In other words, the switching costs were modulated by the cue-to-stimulus intervals, and specifically, switching costs decreased when the preparation time increased. Another intriguing finding was that even when participants were offered enough time to fully prepare for selecting the target language at the cue window, the switching costs were not completely eliminated. This phenomenon reflects what has been referred to as “residual switching costs” which are caused by the exogenous control that can only be overcome when the target stimuli are presented (Rogers and Monsell, 1995; Nicholson et al., 2006; Schneider, 2016). Taken together, these patterns of data suggest that active preparation at the cue window only enables bilinguals to overcome the so-called “endogenous” component of inhibitory control but leaves the “exogenous” control process unresolved until the presence of target stimuli, which in turn leads to the “residual switching costs.” In addition, in the passive dissipation condition, switching costs could be modulated by response-to-cue intervals, that is, switching costs reduced as the dissipation time increased, suggesting that inhibitory control of the non-response language decreased over time after the target language was named.

This beneficial effect of longer over shorter preparation time was reflected in both faster naming latencies and higher accuracy rates. It could be presumably argued that with a short cue-to-stimulus interval (700 ms) in switch trials, advanced language-set reconfiguration of the upcoming trial can not be completed due to the restricted preparation time, while with longer preparation time (1,400 ms) the preparation interval was sufficient to prepare for the reconfiguration process, thus leading to performance improvement. These patterns of data replicate the traditional preparation effects consistently observed in the task switching (e.g., Altmann, 2004; Koch and Allport, 2006) and language switching literature (e.g., Philipp et al., 2007; Fink and Goldrick, 2015; Festman and Mosca, 2016).

However, in spite of the ample preparation (i.e., 1,400 ms in the long preparation condition), the switching costs persist, suggesting that longer active preparation time only enables the participants to overcome the inhibitory control to select the target language at the cue window but cannot fully eliminate the inhibition. These further reveal that some other components of the language switch or inhibitory control can only be completed after the stimulus is presented, referring to as the so-called “residual switching costs.” Furthermore, these patterns of results thus lead the author to argue that there may be distinct control processes in the cued language switching process that could take place (1) following the presentation of the cue and (2) following the presentation of the stimulus. This argument seems to be reasonable, given the two-stage task switching model in which Rogers and Monsell (1995) argued that task-set reconfiguration control is both endogenously and exogenously driven (see also Meiran, 2000 for a similar conclusion).

The observation of a preparation benefit effect on the L1-repeat trial challenges Verhoef et al. (2009) “L1-repeat benefit” theory in which they argued that the activation of the L2 is highly limited during the L1 production, thus leading to the null preparation facilitation effect in L1-repeat trials. In their study, Dutch-English bilinguals performed cued language switching tasks with either shorter or longer cue-to-stimulus intervals of 500 ms and 1,250 ms, respectively. The participants showed asymmetrical switching costs (i.e., larger in Dutch than in English) in the shorter preparation condition, while symmetrical switching costs with longer preparation. The naming latency difference between L1 stay and switch trials reduced when more preparation time was given, since preparation only facilitated the L1-switch trial naming. In contrast, the RT difference between L2 stay and switch trials remained constant, thus making L1 switching costs equivalent to those in the L2 in the longer preparation condition. Given that benefits of preparation are due to the task-set reconfiguration process or inhibition of the non-target language (transient task-set inertia theory), the lack of L1-repeat preparation benefits thus reveals a failure to activate L2 in the L1 production process. Since L2 lexical representations are not activated when producing in L1, there is no need to inhibit them, hence no preparation benefit in L1-repeat trials, in other words, the preparation time to select the L1, as shown in cue-to-stimulus intervals, should not modulate L1 naming latencies. However, this is not the case in this study and the fact that L1-repeat trials also benefited from preparation provides support for the bilingual language co-activation theory and suggests that cross-language activation also operates on L1 repeat trials.

Regarding the interaction between preparation and language, it was found that cue-to-stimulus intervals affected L1 and L2 naming latencies differently, suggesting that L1 and L2 naming latencies benefited unequally as preparation time increased. This result is congruent with previous studies in which researchers consistently observed evidence of greater preparation facilitation for dominant L1 compared to the weaker L2 (e.g., see Fink and Goldrick, 2015; Festman and Mosca, 2016 for a similar observation). This greater L1 preparation facilitation effect can be arguably explained by assuming that the speed of preparing for task reconfiguration is correlated to language proficiency levels, i.e., the more dominant the language, the more effective the preparation process. However, the mechanism of such a relationship between proficiency levels and preparation effect remains unclear. This result also provides compelling support for the “global slowing L1” phenomenon that the L1 lexical representations are globally inhibited compared to those of L2, thus when there is a preparation effect, L1 lemmas would benefit more. Another intriguing finding is that the preparation time benefit was greater for switch trials than for non-switch trials, as reflected by the smaller switching costs in the long preparation condition, which is not a novel finding in the task-switching literature. Again, this suggests that inhibition processes or task-set reconfiguration processes mainly occur on the switch trials and thus allows for a more effective preparation benefit.

While cue-to-stimulus intervals reflect active preparation, language-set dissipation time is related to the interval between “naming response on Trial N-1 and the presentation of the cue for the target stimulus on Trial N,” the response-to-cue intervals (Meiran, 2000, p. 211). In line with the previous literature on task-switching (e.g., Rogers and Monsell, 1995; Meiran, 2000), the results revealed that switching costs could be modulated by response-to-cue intervals – the time for passive dissipation of the language set inhibitory control in the previous trials. In other words, the switching costs decreased as the response-to-cue intervals increased. As argued earlier, Rogers and Monsell (1995) dual-control models make a distinction between two types of control in cued task-switching paradigms – endogenous and exogenous control, and the latter is triggered by the presence of the external stimulus. Therefore, these patterns of results suggest that a longer dissipation time allows the participants to overcome the exogenous component of the inhibitory control process. However, it is shown that the switching costs did not fully disappear, even though a longer passive dissipation time was offered (i.e., 1,400 ms). This could lead one to assume that the long response-to-cue intervals employed in the experiment (i.e., 1,400 ms) were insufficient for the participants to prepare or overcome the exogenous or residual inhibition of the non-target languages.

However, this may not be the only possibility. Remember that when manipulating the cue-to-stimulus intervals in the active preparation condition, the shorter active preparation time (600 ms) proved to be insufficient for the participants to overcome the endogenous part of the inhibitory control process followed by the cue onset, as the switching costs were found to decrease as the cue-to-stimulus intervals increased in the long preparation block. Therefore, it could be argued that in the passive dissipation condition in which the response-to-cue intervals were kept the same as the ones in active preparation condition, the “unfinished” part of endogenous control following the cue-to-stimulus intervals is highly likely to be further transferred to exogenous control, in other words, the part of endogenous control that is not fully overcome continues to play a role after stimulus onset. Consequently, these compound inhibitory control processes result in residual switching costs even though the longer dissipation time was provided. However, both those assumptions are mainly dependent on the author’s intuition and lack the support from the empirical studies, thus further research could focus on examining whether the switching costs can be eliminated when the participants are able to fully prepare in both cue-to-stimulus intervals and response-to-cue intervals.

As argued in the literature review, the locus of the switching costs in bilingual language switching remains unknown. The results observed in active preparation and passive disputation conditions might shed some light on this question and provide support for the assumption that switching costs might occur at two possible loci: (1) a language task schema competition phase in which the intention of naming an object in L1 vs. L2 competes after the cue onset; and (2) a lemma selection phase in which co-activated L1 and L2 lexical representations compete for further speech production (see also Zhu et al., 2018 for a similar argument). However, recent ERP studies on language switching appear to challenge such an assumption since they found that switching costs mainly occur at the lemma selection stage (Guo et al., 2013; Chang et al., 2016). For example, Guo et al. (2013) conducted an ERP study that aimed to examine the loci of inhibition during trilingual word production using the language switching paradigm. The cue-locked ERPs showed marginally significant n-2 repetition effects, whereas the stimulus-locked ERP data showed that a greater negative ERP component was elicited by n-2 repetition trials than by n-2 switch trials around 250 ms after stimulus onset, which is similar to the N2 component, as the index of inhibition of the non-target language, which has been reported in previous ERP studies on bilingual language production. Taken together, the authors argued that inhibition of non-target languages occurs during the lemma-selection stage but not at the language task schema competition stage.

The other evidence that switching costs occur at the lemma selection phase comes from Chang et al. (2016), in which the participants performed a cued language switching task in which cues were presented prior to the stimulus and a modified version in which the target stimulus was presented before the cue, while their electrophysiological responses were recorded by ERPs. The ERPs related to the cue and stimulus for two presentation sequences showed that in the stimulus-cue manipulation, the digit stimulus elicited a negative ERP component around 200 ms, while the cue-locked data revealed a significantly reversed switching costs, that is, ERPs were more negative for repeat trials than for switch trials. In the cue-stimulus sequences, the ERPs time-locked to cues did not show significant effects of switching, whereas the stimulus-locked ERPs revealed an N2 component, with the mean amplitude for the switch trials being more negative than that for repeat trials. Taken together, this pattern of results led them to argue that switching costs mainly occurred at the lemma selection phase, replicating Guo et al. (2013) findings.

At first glance, these two studies seem to provide compelling evidence against the assumption in the present study that there are two possible loci for switching costs. However, given that the stimuli used in these two studies are different from those in the current study (i.e., Arabic digits in the two ERP studies and pictures in the present study), it may be argued that these manipulations make it impossible to compare directly across studies. Furthermore, the results observed by Declerck et al. (2012) clearly demonstrated that the digit stimuli allow for a reduction in switching costs, which leads the writer to argue that digits have a potential effect on the control process at the cue window (or the language task schema repetition stage). Specifically, this pre-stimulus language control could possibly involve a preparation bias for the target language, enabling the participants to prepare a set of digits in the target language and/or suppress those in the non-target language. In other words, naming the very limited number of digits may not require cognitive control or language competition during the language task scheme stage, instead it only involves the selection of a certain set of digits in the target language. Consequently, it might be the case that those authors did not find switching effects following cue onset. Furthermore, one weaker version of this argument might be that the suppression of non-target language digits is much weaker than whole-language control, and thus can not be fully detected by ERPs. At the very least, naming the limited number of digits does not necessarily require the inhibitory control of the whole non-target language but only certain digit sets of the non-target language.

In sum, it is still not entirely clear where language control occurs, and the precise role of different stages remains unknown. Nevertheless, it is clear that there is more than one stage involved in bilingual language control. For example, language control could occur at the language processing stage after the stimulus onset, i.e., at the concept level, the lemma level and even sometimes the phonological level. In addition, language control could also occur outside the language processing stage at the language task schema level. Therefore, proficient bilinguals would have a greater ability to control the language task schema, deciding which language to be responded, which supports the argument that language switching ability can lead to an improvement in cognitive functions such as executive control, known as a bilingual advantage (e.g., Prior and MacWhinney, 2010; Prior and Gollan, 2011; Soveri et al., 2011; Wu et al., 2016). Therefore, future research should aim to investigate the relationship between different stages of language control and how they relate to executive control or control in general.

## 7. Limitations

As noted earlier, there was not a systematic questionnaire or interview conducted in the current study to collect information regarding participants’ family language use, the frequencies of second language use, and how often they switch between Chinese and English, which are factors that can affect bilinguals’ switching performance and their inhibitory control ability (e.g., Liu et al., 2019). Therefore, future research on language switching needs to consider the utilisation of such a questionnaire, i.e., the LEAP-questionnaire (Kaushanskaya et al., 2020) to control the effects of those variables on results. Additionally, although participants’ IELTs scores and self-report interviews were used to indicate proficiency, this is inadequate, and a standard test such as the Multilingual Naming Test (Gollan et al., 2012) should be considered to capture participants’ lexical robustness and language proficiency statistically in future work. Secondly, as noted earlier, it is argued that inhibitory control cannot be fully completed until the stimulus is presented because switching costs were observed even though the preparation time (indexed by the cue-to-stimuli interval) was prolonged from 600 to 1,400 ms. However, considering that only two preparation times were given in the cue-to-stimulus interval, it might be inadequate to make such a strong claim as if the preparation is long enough, such as more than 8,000 ms, inhibitory control might be finished at the cue-to-stimulus interval.

## Data availability statement

The raw data supporting the conclusions of this article will be made available by the authors, without undue reservation.

## Ethics statement

The studies involving human participants were reviewed and approved by Department of Theoretical and Applied Linguistics Ethics Committee (DTAL) at the University of Cambridge. The patients/participants provided their written informed consent to participate in this study.

## Author contributions

QS designed and conducted the study, completed the statistical analysis, and wrote the manuscript.

## Conflict of interest

The author declares that the research was conducted in the absence of any commercial or financial relationships that can be construed as a potential conflict of interest.

## Publisher’s note

All claims expressed in this article are solely those of the authors and do not necessarily represent those of their affiliated organizations, or those of the publisher, the editors and the reviewers. Any product that may be evaluated in this article, or claim that may be made by its manufacturer, is not guaranteed or endorsed by the publisher.
