# Supplementary material for: Investigating the modulation of active preparation and passive dissipation on inhibitory control processes in the language switching paradigm
Source: Front Psychol. 2023 Jan 27;14:1065268. doi: 10.3389/fpsyg.2023.1065268 (PMC9911414; doi:10.3389/fpsyg.2023.1065268)
Supplement: Supplementary file 1 [file Data_Sheet_1.docx]

**Appendix:**

All picture stimuli used in the current study comes from: https://crl.ucsd.edu/experiments/ipnp/

Experimental paradigm

Figure 3A. Paradigm of active preparation conditions (short preparation and long preparation, respectively)


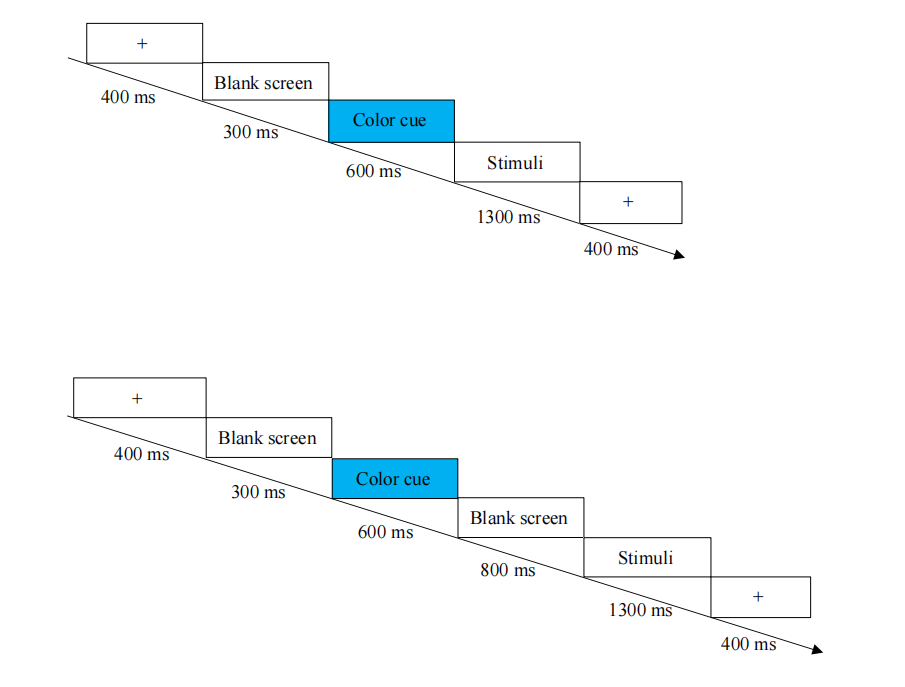


Figure 3B. Paradigm of passive preparation conditions (short preparation and long preparation, respectively)


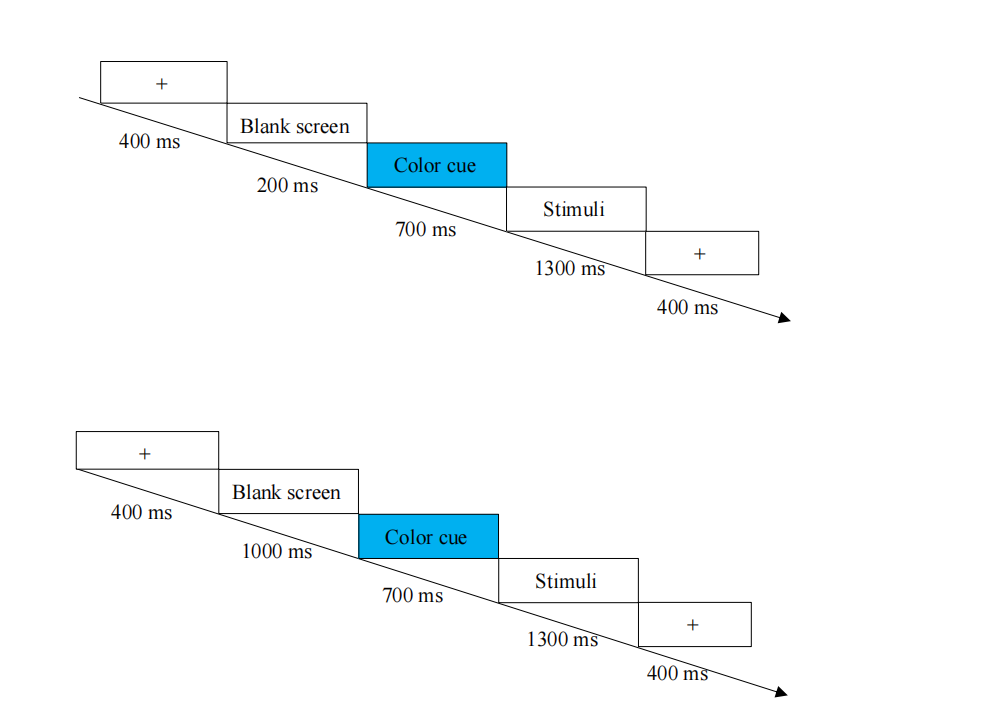


Table 3. Trial sequence

| Block | Trial sequence |
| --- | --- |
| Short active preparation block | L2, L1, L1, L1, L2, L2, L2, L1, L2, L1, L1, L2, L1, L2, L1, L1, L2, L2, L2, L2, L2, L1, L2, L2, L1, L1, L1, L2, L1, L1, L2, L1, L2, L1, L1, L1, L1, L2, L2, L2, L2. |
| Long active preparation block | L2, L1, L1, L1, L2, L1, L1, L2, L1, L2, L1, L1, L1, L1, L2, L2, L2, L2, L2, L1, L1, L1, L2, L2, L2, L1, L2, L1, L1, L2, L1, L2, L1, L1, L2, L2, L2, L2, L2, L1, L2 |
| Short passive preparation block | L1, L2, L2, L2, L1, L2, L1, L1, L2, L1, L2, L1, L1, L2, L2, L2, L2, L2, L1, L2, L2, L1, L1, L1, L2, L1, L1, L2, L1, L2, L1, L1, L1, L1, L2, L2, L2, L2, L1, L1, L1 |
| Long passive preparation block | L1, L2, L1, L1, L2, L1, L2, L1, L1, L1, L1, L2, L2, L2, L2, L1, L1, L1, L1, L2, L2, L2, L1, L2, L1, L1, L2, L1, L2, L1, L1, L2, L2, L2, L2, L2, L1, L2, L2, L1, L1 |
